# Gut Microbiota Metabolic Reprogramming Drives Endocrine and Immune Resistance in Hormone-Dependent Cancers

**DOI:** 10.3390/cancers18081218

**Published:** 2026-04-12

**Authors:** Zhengqin Zhu, Yiting Yang, Libin Pan, Liefeng Ma, Luo Fang

**Affiliations:** 1 College of Pharmaceutical Science, Zhejiang University of Technology, Hangzhou 310022, China; 2 Department of Pharmacy, Zhejiang Cancer Hospital, Hangzhou Institute of Medicine (HIM), Chinese Academy of Sciences, Hangzhou 310022, China; 3 Department of Pharmacy, Zhejiang Chinese Medical University, Hangzhou 310022, China; 4 Department of Hepatobiliary and Pancreatic Surgery, Zhejiang Cancer Hospital, Hangzhou Institute of Medicine (HIM), Chinese Academy of Sciences, Hangzhou 310022, China; 5 Postgraduate Training Base Alliance of Wenzhou Medical University (Zhejiang Cancer Hospital), Hangzhou 310022, China

**Keywords:** gut microbiota, hormone-dependent malignancies, estrobolome, androbolome, tumor microenvironment

## Abstract

The gut microbiota, acting as a critical extrinsic endocrine organ, is profoundly involved in the pathological evolution and therapeutic response of hormone-dependent malignancies. This review elucidates the core mechanisms governing the microbiota, endocrine, and immune triple-axis. Multi-omic and biochemical evidence demonstrates that microbial metabolic networks, comprising the estrobolome, androbolome, and progestobolome/corticobolome, rely on enzymatic systems such as β-glucuronidases (GUS) and steroid-17,20-desmolases to execute hormone deconjugation and structural modification, thereby modulating systemic steroid exposure. Concurrently, microbe-derived metabolites, such as secondary bile acids and purine derivatives, act as inter-kingdom messengers. These metabolites remodel the tumor immune microenvironment by antagonizing hormone receptors and activating specific signaling axes, such as the Inosine-A2AR pathway. By modulating localized immune cells like effector T cells and myeloid cells, they play a pivotal role in tumor immune evasion. Furthermore, pharmacomicrobiomics reveals a bidirectional regulation between anti-tumor agents and the gut microbiota, where endocrine and immunotherapeutic drugs can induce microbial dysbiosis, while specific gut taxa contribute to primary or acquired resistance by enzymatically inactivating drugs (e.g., reductive inactivation of Enzalutamide) or providing hormonal precursors through bypass pathways. Facing translational challenges, such as real-world microbiome complexity and the colonization resistance of indigenous flora, we propose treating the human body as a unified host–microbe holobiont system. Future research should leverage gnotobiotic models and genetic causal inference to establish functional causality. These efforts will facilitate the development of precision tools, including ubiquitin–proteasome system (UPS) modulators, microbial enzyme inhibitors, and engineered live biotherapeutics. Collectively, these systems biology strategies offer a robust framework for overcoming therapeutic resistance in hormone-dependent malignancies.

## Simple Summary

Clinical management of hormone-dependent malignancies, such as breast and prostate cancers, is frequently challenged by therapeutic resistance and inter-individual variability in treatment efficacy. As a critical extrinsic endocrine organ and a systemic metabolic reactor within the human body, the gut microbiota plays a pivotal role in the pathological progression of these tumors. This review synthesizes the core causal mechanisms by which the gut microbiota reshapes the tumor microenvironment through enzymatic modulation of hormone metabolism and anti-tumor immunity. Furthermore, we provide an in-depth analysis of how drug-microbe interactions influence the efficacy and toxicity profiles of anti-cancer pharmacological agents. Within this framework, deciphering the systemic interactions among the host, tumor, and microbiota is expected to offer novel perspectives for overcoming endocrine resistance. Future interventional strategies, encompassing precision nutrition, postbiotics, and engineered microbial consortia, represent vital translational pathways for optimizing clinical outcomes in cancer patients.

## 1. Introduction

Traditional endocrinology has long perceived the metabolism of sex hormones as a host-centric, closed-loop process. In this conventional model, hormones are synthesized in the gonads or adrenal glands, biochemically transformed in the liver, and ultimately eliminated via the kidneys. However, this host-centric paradigm fails to account for the profound clinical heterogeneity observed in oncology. Even among patients with hormone-dependent tumors who possess normal organ function and similar pathological profiles, standard dosages of endocrine therapies, such as the aromatase inhibitor exemestane [1] or the selective estrogen receptor modulator tamoxifen [2,3], yield markedly different levels of systemic hormone exposure, drug bioavailability, and anti-tumor efficacy [4].

With the expansion of multi-omic technologies, the indispensable role of the gastrointestinal and extra-intestinal microbiota in maintaining endocrine homeostasis has come to light. The gut is no longer viewed merely as an organ for nutrient absorption and waste excretion, but rather as a niche for microbial communities that function as a systemic extension of the tumor microenvironment [5,6]. This microbial ecosystem encodes a vast repertoire of metabolic enzymes capable of executing biochemical modifications that the host genome lacks [7,8]. Emerging evidence indicates that the gut microbiota constitutes an auxiliary regulatory network for sex hormone metabolism, operating beyond the classical target-organ axes [9,10,11]. Through microbe-mediated structural modifications, specific gut taxa act as pivotal gatekeepers of systemic steroid homeostasis by leveraging a diverse repertoire of microbial enzymes, thereby influencing the pharmacological disposition of endocrine therapies [3,4]. This host-microbial co-metabolism network dictates the systemic exposure of targeted therapies and modulates the local tumor immune microenvironment, specifically through the endocrine regulation of immune cell activity. Consequently, the microbiome has emerged as a critical determinant in the initiation, progression, and therapeutic sensitivity of breast, prostate, and endometrial cancers [4,12,13].

The present review aims to decipher this intricate host-microbial co-metabolic landscape. We will explore the key enzymatic mechanisms by which the microbiota regulates steroid and drug metabolism, focusing on specialized functional branches, such as the estrobolome and androbolome. Furthermore, we analyze how synchronized dysbiosis across anatomical sites contributes to acquired resistance via the microbiota-endocrine-immune axis [14,15]. Building upon these insights, we systematically evaluate multi-faceted intervention strategies encompassing precision nutrition, natural products, and live biotherapeutics. Our objective is to establish a translationally valuable theoretical framework for the precision companion diagnosis and systemic ecological intervention of hormone-dependent malignancies [8,10].

## 2. Underlying Biochemical Mechanisms of Microbial Intervention in Host Sex Hormone Metabolism

### 2.1. Expansion of Sex Hormone Modification Pathways via Microbial Deconjugation and Structural Transformation

**Figure 1:**
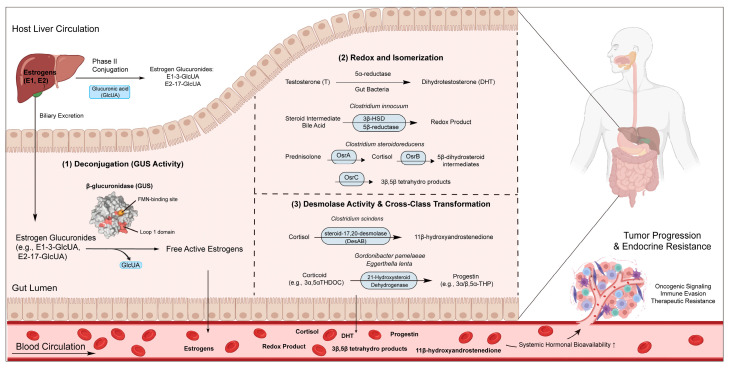
The systemic microbiota–liver–tumor axis in sex hormone metabolism. The intestinal ecosystem serves as a central hub in the enterohepatic circulation (EHC) and systemic metabolism of host hormones, operating through three major functional nodes: (1) Deconjugation: Microbial β-glucuronidases (GUS), particularly those harboring specialized structural motifs such as Loop 1, hydrolyze hepatic-derived estrogen-glucuronide conjugates to liberate biologically active free estrogens. (2) Redox and Isomerization: Diverse gut taxa modify the steroid scaffold via enzymatic pathways, including the 5α-reductase pathway for testosterone-to-DHT conversion and the OsrABC multi-enzyme complex for corticosteroid reduction. (3) Cross-Class Transformation: Commensal species (e.g., *C. scindens*) utilize steroid-17,20-desmolases (DesAB) to cleave side chains, shunting glucocorticoids into 11-oxy-androgens. Collectively, these microbial transformations increase the systemic bioavailability of active steroids, which are reabsorbed into the blood circulation and subsequently infiltrate the tumor microenvironment to drive oncogenic signaling, immune evasion, and adaptive endocrine resistance.

Traditional physiology typically defines the host liver as the metabolic terminus for sex hormones. In this canonical pathway, the liver converts free hormones into water-soluble conjugates through glucuronidation or sulfation, which are subsequently excreted via bile into the gut or eliminated through urine. However, the gut microbiota, leveraging its extensive genomic repertoire, expands this unidirectional clearance route. By expressing specialized enzymatic systems, the gut microbiota establishes a systemic microbiota–liver–tumor metabolic axis. The core functions of this operational logic rely on three critical microbial metabolic nodes that dictate the systemic bioavailability of active steroids (Figure 1):

(1) Deconjugation-mediated hormone liberation: During hepatic metabolism, estrogens are conjugated with glucuronic acid to enhance solubility. Gut microbial β-glucuronidases (GUSs) selectively hydrolyze these conjugates back into free estrogens [16,17]. This process is driven by enzymes from diverse human gut microbes, particularly those harboring specialized structural motifs such as Loop 1, mini-Loop 1, and FMN-binding domains, which dictate the deconjugation efficiency of estrone-3-glucuronide (E1-3-G) and estradiol-17-glucuronide (E2-17-G). Consequently, microbial GUS activity serves as a primary biochemical switch that dictates the equilibrium between conjugated and free steroids within the intestinal lumen.
(2) Redox and isomerization of the steroid scaffold: In addition to hydrolysis, the gut microbiota extensively participates in the direct modification of the steroid nucleus. Certain intestinal taxa express 5α-reductases, which facilitate the stereospecific reduction of the Δ^4^ double bond in the steroid A-ring, thereby converting testosterone into its more potent metabolite, dihydrotestosterone (DHT) [18]. Furthermore, specific strains such as *Clostridium innocuum* harbor genes for 3β-hydroxysteroid dehydrogenase (3β-HSD) and 5β-reductase, which are involved in the redox reactions of bile acids and steroid intermediates [19]. Under conditions of acute immune stress, the gut microbiota can also modulate local intestinal corticosterone production and influence the levels of its 3α/3β- and 5α-reduced metabolites [20]. Recent studies have identified various bacteria, such as *C. steroidoreducens*, that directly reduce cortisol via the OsrABC multi-enzyme complex, further diversifying the microbial redox landscape [21,22].
(3) Cross-class transformation via carbon-chain cleavage: The gut microbiota not only modifies individual hormones but also mediates the inter-conversion between different hormonal classes. Biochemical characterizations reveal that commensal species, such as *C. scindens*, express a steroid-17,20-desmolase (DesAB) [23]. This enzyme directly cleaves the side chains of glucocorticoids, including cortisol and prednisolone, converting them into 11-oxy-androgens such as 1,4-androstadiene-3,11,17-trione [23,24]. Simultaneously, taxa like *Gordonibacter pamelaeae* and *Eggerthella lenta* utilize 21-dehydroxylases to shunt glucocorticoids into progestins [25,26]. Collectively, these microbe-driven structural modifications and cross-class conversions transform the gut into a metabolic reactor. As illustrated in Figure 1, the resulting active steroids are reabsorbed into the blood circulation, significantly increasing systemic hormonal bioavailability. This systemic influx subsequently infiltrates the tumor microenvironment (TME), providing the essential fuel to drive oncogenic signaling, immune evasion, and adaptive endocrine resistance.

### 2.2. Specific Microbial Metabolic Networks Constitute the Enzymatic Basis for Sex Hormone Transformation

To systematically decipher complex microbial enzymatic networks, the academic community has introduced specific microbial metabolic concepts, notably the estrobolome and androbolome [10,27]. These frameworks aim to elucidate the microbial gene sets and core enzymatic repertoires that drive specific sex hormone metabolism from a metagenomic perspective.

The estrobolome encompasses the aggregate of microbial genes and their encoded enzymatic systems within the gut that possess estrogen-metabolizing potential [27]. The biochemical driving force of this functional landscape primarily originates from the highly heterogeneous GUS family [16,17]. Multi-omic data reveal significant inter-individual variability in the abundance and expression patterns of GUS-encoding genes across different hosts. This enzymatic heterogeneity objectively influences the efficiency of the EHC of conjugated estrogens, thereby modulating systemic estrogen exposure [3,28]. The metabolic plasticity of the estrobolome is governed by the structural diversity of GUS isoforms, which possess varying affinities for different estrogenic sub-types. This enzymatic heterogeneity determines the flux of deconjugated steroids available for re-entry into the systemic pool, thereby establishing a microbial-driven set point for endocrine homeostasis.

The androbolome delineates the dynamic equilibrium of androgen synthesis, transformation, and degradation mediated by the gut microbiota. On one hand, this network includes microbial taxa with inter-kingdom synthetic potential, such as *C. scindens*, which harbors genes encoding steroid-17,20-desmolases [23,24,29]. These bacteria can operate independently of the host endocrine axis to cleave glucocorticoids, such as cortisol, into 11-oxy-androgens like 1,4-androstadiene-3,11,17-trione. By bypassing the canonical host steroidogenic pathways, this microbial enzymatic bypass expands the pool of C19 steroids within the intestinal environment. Such independent structural transformations highlight the capacity of the androbolome to generate biologically active androgens from non-gonadal precursors through carbon-chain cleavage. On the other hand, the androbolome encompasses pathways for androgen degradation and biotransformation. For instance, *Pseudomonas nitroreducens* [30] utilizes specialized dehydrogenases to execute the catabolic degradation of testosterone, exhibiting a high substrate specificity for the steroid nucleus under lipid-rich microenvironments. Similarly, specific commensals, such as *Thauera* sp., can reduce host serum androgen levels by intervening in the EHC of these steroids [31]. Additionally, high 5α-reductase activity in certain taxa, such as *Odoribacter splanchnicus*, positively correlates with elevated dihydrotestosterone-to-testosterone (DHT/T) ratios in females [18]. These insights suggest that the androbolome exerts bidirectional regulatory effects on the homeostasis of the systemic androgen.

The progesto/corticobolome serves as a molecular hub mediating the transformation of C21 steroid precursors and the crosstalk within stress networks. Biochemical evidence demonstrates that commensal bacteria, such as *C. scindens*, can perform reductive metabolism on glucocorticoids like cortisol to generate metabolites such as 5α-dihydrocortisol, thereby modulating the host steroid [24]. During acute immune stress, the gut microbiota can intervene in the hormonal output of the hypothalamic-pituitary-adrenal (HPA) axis by utilizing oxidoreductase pathways for the extensive reductive transformation of corticosterone and progesterone, ultimately impacting systemic immune and stress homeostasis [20]. Recent mechanistic studies have revealed even more refined metabolic pathways, gut bacteria can directly reduce cortisol via the OsrABC multi-enzyme complex [21,22], while certain microbes express 21-dehydroxylases that shunt glucocorticoids into progestins [25,26]. Furthermore, the apmAB reductase encoded by *Clostridium innocuum* is responsible for converting progesterone into epipregnanolone, a metabolite with minimal biological activity, leading to the direct inactivation of host progesterone.

The underlying redox networks support the biochemical operation of the aforementioned specialized hormone-bolomes. This network is exemplified by taxa expressing specific dehydrogenases and reductases, such as *C. innocuum* [19,32]. These microbes are responsible for processing intermediates in various steroid metabolic pathways and participate in the transformation network of primary bile acids into secondary bile acids, such as deoxycholic acid [32]. The stability of this basal redox network links host lipid metabolism and local intestinal inflammation with the sex-hormone microbial network, collectively maintaining systemic endocrine balance [19]. To illustrate the complexity of this host-microbial co-metabolism, we have systematically summarized the currently validated microbial taxa, specialized enzymatic systems, and their driven endocrine phenotypes based on the latest enzymatic identifications and multi-omic evidence (Table 1).

### 2.3. Microbially Mediated Enterohepatic Circulation Directly Dictates the Systemic Bioavailability of Targeted Hormones

The extent of biochemical modification exerted by the gut microbiota on the host endocrine system depends not merely on the catalytic efficiency of individual enzymatic reactions, but rather relies heavily on the enterohepatic circulation (EHC) as a core physiological hub. From a systems biology perspective, the kinetic parameters of bacterial metabolic enzymes during single catalytic events, such as the Michaelis constant (Km) or maximum velocity (Vmax) for GUS-mediated deconjugation [17] or DesAB/OsrABC-catalyzed cross-class transformations, typically do not exhibit significant kinetic advantages. However, the gut microbiota generates a cumulative amplification effect within the host through high-frequency EHC. Subtle inter-host variations in microbial enzymatic activity are progressively magnified within the recurrent, closed loop of biliary excretion and intestinal mucosal reabsorption. This circulatory amplification implies that even marginal differences in microbial catalytic capacity can be exponentially leveraged over time. From a kinetic perspective, the gut acts as a biological reservoir where microbial enzymes continuously reshape the steroid pool, thereby dictating the systemic availability of active moieties independently of host hepatic output. A similar amplification effect exists within the androgen metabolic network. Specific commensals, such as the GDN1 strain of *Thauera* sp., effectively reduce host systemic serum androgen levels by selectively intercepting the biochemical recycling of androgens within the EHC [31]. Conversely, specific taxa with synthetic potential can leverage this circulatory pathway to provide a continuous supply of alternative androgens through microbe-mediated structural modifications and bypass compensation, thereby providing a sustained influx of active ligands that may circumvent traditional host-mediated endocrine suppression. Consequently, the amplification effect rooted in EHC provides a kinetic explanation for why patients with comparable organ function and tumor staging exhibit marked heterogeneity in serum hormone levels and clinical phenotypes. This mechanism offers vital biochemical clues for deciphering primary or acquired resistance to targeted endocrine therapies.

## 3. Co-Regulation of the Hormone-Dependent Tumor Microenvironment by the Gut Microbiota and the Endocrine Axis

### 3.1. Mechanisms of Systemic Immune Suppression Mediated by Hormonal Signaling Pathways

Beyond regulating systemic hormone exposure, the gut microbiota serves as a mechanistic rheostat for the tumor microenvironment (TME) by modulating the hormone-immune crosstalk [10]. Building upon the principles of immunometabolism, evidence indicates that sex hormone receptors are extensively expressed across diverse immune cell populations, establishing an immunomodulatory network indirectly orchestrated by microbial metabolic outputs [33]. This hormone-immune crosstalk establishes an immunomodulatory network that is indirectly regulated by the gut microbiota. Specifically, the immunological regulation dictated by hormones and the microbiota exhibits distinct context-dependency and sexual dimorphism. In systemic non-hormone-driven cancers, such as colorectal cancer, estrogen signaling typically exerts protective effects by mitigating local intestinal inflammation and ameliorating the immunosuppressive state of the TME. Unique microbial structures in female hosts, characterized by variations in specific GUS activities, can increase the proportion of tumor-infiltrating effector T lymphocytes and enhance their cytotoxic killing capacity [34,35]. Conversely, in hormone-dependent tumors such as breast cancer, excessive estrogen exposure mediated by microbial dysbiosis often induces localized immune tolerance. On the other hand, androgen signaling generally performs a negative immunomodulatory role within the TME. Mechanistic insights reveal that endogenous androgen receptor (AR) signaling in T cells directly represses the transcriptional program of interferon-γ (IFN-γ), ultimately leading to the functional exhaustion of CD8+ T cells [13]. During this process, specific microbe-derived secondary metabolites serve as critical inter-kingdom messengers. For instance, specific microbe-derived secondary bile acids function as active inter-kingdom messengers. By traversing the mucosal barrier and infiltrating the TME, these metabolites competitively antagonize the AR on the surface of host immune cells, thereby actively reprogramming the hormone-mediated immunosuppressive network to re-sensitize the tumor to anti-tumor immunity [12]. Crucially, this microbial-hormone-immune crosstalk not only modulates effector cytokine production but also directly participates in the multi-level fine-tuning of PD-1 expression, which constitutes a fundamental molecular basis for immune evasion in hormone-dependent cancers [36,37,38]. These findings provide a definitive causal link suggesting that targeting the microbiota-endocrine-immune axis is a robust strategy for overcoming therapeutic resistance.

### 3.2. Metabolic Characteristics of Local Microbiota Dictate the Pathological Evolution of Specific Cancer Types

The progression of breast cancer is intricately linked to the biological significance of estrobolome imbalance. Clinical observations indicate that as ovarian hormone secretion declines in postmenopausal hosts, the gut microbiota undergoes systemic alterations characterized by decreased overall diversity and shifts in the abundance of specific GUS enzymes [39]. To establish definitive causality, studies using germ-free (GF) mouse models have demonstrated that the transplantation of an estrobolome-rich microbiota actively drives mammary epithelial proliferation by elevating circulating estrogen levels [40]. This sustained hyperestrogenism further reshapes the local immune microenvironment. It not only upregulates PD-1 expression, leading to CD8+ T cell exhaustion, but also increases the infiltration of immunosuppressive regulatory T cells (Tregs), driving the tumor toward an immunologically cold phenotype [41]. In patients resistant to aromatase inhibitor (AI) therapy, an increased abundance of the intestinal genus *Veillonella* has been observed, suggesting a potential role for the microbiota in acquired endocrine resistance [1].

The advancement of prostate cancer exemplifies the microbial bypass within the androgen axis. This microbial enzymatic bypass provides alternative androgenic stimulation to prostate cancer cells, serving as a decisive factor in the emergence of castration resistance (CRPC) [23,29]. Similar cross-talk is evident in polycystic ovary syndrome (PCOS), where microbially driven bile acid shifts orchestrate androgen excess [42]. This microbe-mediated synthesis not only provides non-gonadal hormonal sources but also generates bypass signals that act on tumor-associated macrophages (TAMs). This interaction induces TAM polarization toward an M2-type immunosuppressive phenotype, characterized by high CD206 expression and IL-10 secretion, thereby facilitating resistance to androgen receptor signaling inhibitors (ARSI) [43].

The pathogenesis of endometrial cancer is characterized by a triad of metabolic and endocrine interactions. In the context of NCDs, most notably obesity, the chronic depletion of SCFAs triggers a systemic epigenetic void—characterized by altered histone acetylation [44]—that exacerbates metabolic dysfunction and drives abnormal endometrial proliferation [45]. Integrating these models with genetic causal inference frameworks, such as bidirectional Mendelian randomization, is essential to elucidate the complex causal links between the microbiome, its metabolic outputs (e.g., SCFAs), and host oncogenic traits [46]. Furthermore, dysbiosis across anatomical sites, including the gut and vagina, facilitates the translocation of bacterial lipopolysaccharides (LPSs), triggering the local release of pro-inflammatory cytokines such as IL-6 and TNF-α [47,48]. These signals synergize with free estrogens to activate the PI3K/AKT/mTOR signaling pathway, driving pathological evolution [15].

**Figure 2:**
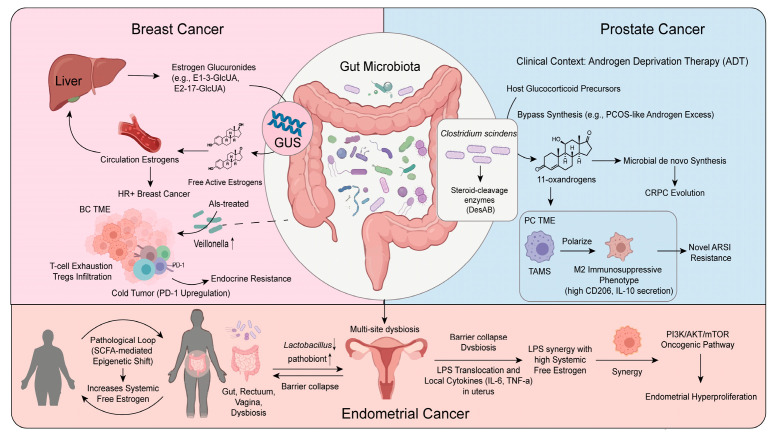
A comprehensive landscape of the mechanisms by which the gut microbiota reshapes the evolution and microenvironment of hormone-dependent malignancies. Breast Cancer: Intestinal GUS enzymes enhance systemic free estrogen levels via EHC, thereby driving the proliferation of HR+ tumors and inducing an immunosuppressive TME, characterized by T cell exhaustion, Treg infiltration and a cold tumor phenotype (PD-1 upregulation). Furthermore, the enrichment of *Veillonella* induced by aromatase inhibitor (AI) therapy promotes acquired endocrine resistance. Prostate Cancer: Specific gut taxa, such as *Clostridium scindens*, utilize DesAB enzymes for the bypass synthesis of 11-oxy-androgens (mimicking PCOS-like androgen excess), which exacerbates CRPC and induces the polarization of TAMs toward the M2 phenotype, ultimately leading to ARSI resistance. Endometrial Cancer: Obesity-driven SCFA depletion triggers a pathological loop (SCFA-mediated epigenetic shift), which synergizes with coordinated dysbiosis across anatomical sites and LPS translocation to activate the PI3K/AKT/mTOR pathway and facilitate abnormal endometrial proliferation. (GUS, β-glucuronidase; HR+, hormone receptor-positive; TME, tumor microenvironment; Tregs, regulatory T cells; ADT, androgen deprivation therapy; CRPC, castration-resistant prostate cancer; TAMs, tumor-associated macrophages; ARSI, androgen receptor signaling inhibitors; LPS, lipopolysaccharides; SCFA, short-chain fatty acids.).

To decipher this complex network, we have constructed a comprehensive landscape of the microbiota-endocrine-immune systemic interaction (Figure 2). Furthermore, the specific molecular mechanisms of these cross-organ interactions are summarized in Table 2.

## 4. The Gut Microbial Network Influences Clinical Responses to Endocrine and Immune-Targeted Therapies

### 4.1. Specific Microbial Enzymatic Systems Modulate the Pharmacokinetics and Adverse Effects of Endocrine Drugs

**Figure 3:**
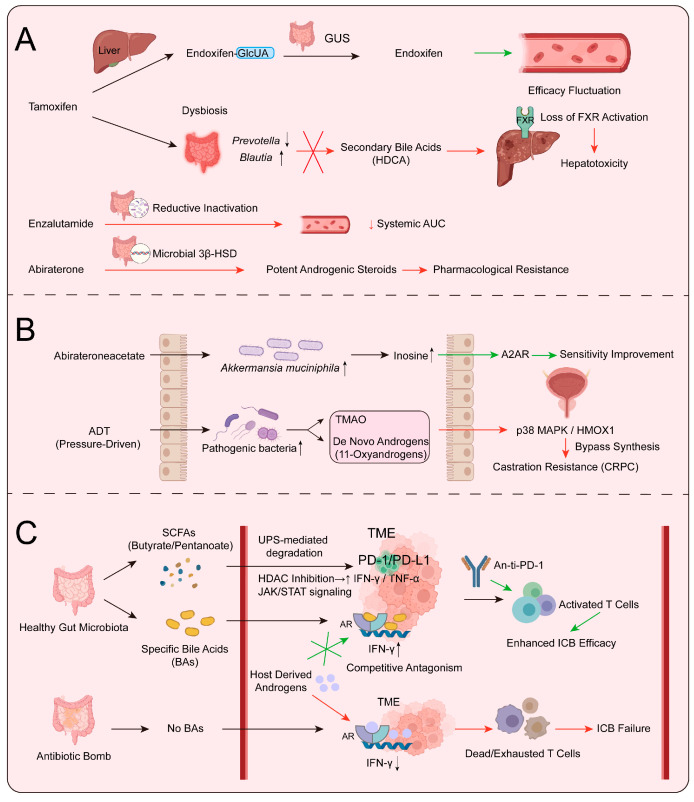
Biochemical and molecular mechanisms of the microbiota–endocrine–immune axis in modulating therapeutic efficacy. (**A**) Pharmacokinetics and Toxicity: The gut acts as a metabolic reactor where microbial GUS enzymes and reductive pathways (for enzalutamide) modulate systemic exposure. Microbial 3β-HSD transforms Abiraterone into potent androgens, driving pharmacological resistance. Drug-induced dysbiosis (e.g., *Prevotella*) disrupts the HDCA–FXR axis, leading to hepatotoxicity. (**B**) Adaptive Resistance: Chronic therapeutic pressure enriches *Akkermansia*, promoting the production of Inosine which enhances sensitivity via the A2AR receptor. Conversely, pathogenic enrichment facilitates 11-Oxyandrogen bypass synthesis and TMAO production, driving p38 MAPK/HMOX1 signaling and CRPC progression. (**C**) ICI Immunotherapy: Healthy microbiota-derived metabolites optimize the TME. SCFAs (Butyrate/Pentanoate) regulate PD-1/PD-L1 stability via UPS-mediated degradation, JAK/STAT signaling, and HDAC inhibition, the latter boosting effector cytokines (IFN-γ). Secondary BAs competitively antagonize AR to alleviate transcriptional repression of IFN-γ, reversing immune evasion. (A2AR, Adenosine A2A Receptor; UPS, Ubiquitin-Proteasome System; 3β-HSD, 3β-Hydroxysteroid Dehydrogenase).

In the clinical practice of targeted oncology, the administered dose of a drug does not invariably equate to the ultimate level of systemic exposure. Following the administration of oral endocrine agents or systemic immunotherapeutic antibodies, these compounds are frequently modulated by biochemical networks established by the gut microbiota. This molecular-level tripartite interaction among the host, drug, and microbes not only dictates bioavailability and adverse toxicities but also provides critical biochemical clues for deciphering the mechanisms underlying widespread primary and acquired resistance. The metabolic fate of tamoxifen in breast cancer therapy exemplifies a canonical pharmacokinetic closed-loop regulated by the microbiota. As a prodrug, tamoxifen requires hepatic transformation via the cytochrome P450 enzymatic system into pharmacologically active metabolites, such as endoxifen. These active moieties are subsequently glucuronidated and excreted into the intestinal lumen via bile [3]. At this juncture, the polymorphism of the gut microbiota emerges as a decisive variable in therapeutic efficacy. Beyond Tamoxifen, the clinical efficacy of second-generation anti-androgens, such as Enzalutamide, is directly modulated by microbial metabolic bypasses. Recent evidence suggests that specific gut commensals facilitate the reductive inactivation of targeted agents, thereby lowering the effective systemic AUC and drug concentration within the TME [14]. Furthermore, the gut acts as a metabolic reactor where microbial 3β-HSD activity transforms Abiraterone into more potent androgenic steroids, creating a state of pharmacological resistance independently of host hepatic metabolism [58,59]. Beyond influencing the exposure of active metabolites, the gut microbiota is implicated in the pathogenesis of endocrine drug toxicities. Longitudinal cohort studies have revealed that tamoxifen treatment significantly alters the microbial architecture, notably through the enrichment of the genus *Blautia* and the depletion of *Prevotella* [60]. Such drug-induced dysbiosis further disrupts the biosynthesis of essential secondary bile acids. For instance, the depletion of hyodeoxycholic acid (HDCA), a bile acid with hepatoprotective properties, inhibits farnesoid X receptor (FXR) signaling, thereby increasing the risk of drug-induced liver injury (DILI) in a subset of patients [61] (Figure 3A).

### 4.2. Endocrine Therapeutic Pressure Induces Gut Microbial Alterations and Promotes Adaptive Resistance

Prolonged endocrine intervention, while effectively blocking host oncogenic signaling, concurrently precipitates structural shifts in the systemic microbiota. This adaptive succession, driven by pharmacological selective pressure, in turn modulates the long-term prognosis of patients. In real-world breast cancer cohorts, adjuvant endocrine therapy (AET) significantly alters the intestinal metabolite pool and microbial abundance. Longitudinal tracking reveals that patients who undergo extensive AET and eventually experience relapse exhibit a gut microbiota characterized by diminished α-diversity and the enrichment of specific genera, such as *Sutterella* and *Ruminococcus*. These distinct colonization patterns emerging under drug pressure have become independent biomarkers for predicting shortened progression-free survival (PFS) and heightened recurrence risk [62,63]. Similarly, investigations into aromatase inhibitors (AI) confirm that the enrichment of opportunistic pathogens, such as *Veillonella*, in resistant patients suggests a ubiquitous role for the microbiota across diverse mechanisms of endocrine resistance [1]. In the context of anti-androgen therapies (ADT/ARSI) for prostate cancer, the interaction between drugs and the microbiota exhibits notable pleiotropy. On one hand, oral administration of abiraterone acetate can enrich *Akkermansia muciniphila*. In the context of immunotherapy, the production of inosine by specific commensals, such as *Bifidobacterium pseudolongum*, has emerged as a critical immunomodulatory mechanism. This microbial-derived purine metabolite activates antitumor T cells by signaling through the adenosine A2A receptor, providing a definitive biochemical link between the microbiome and the efficacy of ICIs [64]. Establishing this causal link via germ-free models underscores that the microbiome is an active driver of therapeutic outcomes rather than a passive observer. On the other hand, the microbial network manifests specific metabolic compensatory features in response to sustained endocrine blockade. During the evolution toward castration-resistant prostate cancer (CRPC), specific gut bacteria not only provide androgenic precursors via microbe-mediated structural bypasses but also generate metabolites, such as trimethylamine N-oxide (TMAO), that traverse the intestinal barrier. These metabolites directly activate the p38 MAPK/HMOX1 signaling pathway, thereby facilitating the proliferation and invasion of prostate cancer cells [14,65,66,67]. This microbial succession triggered by endocrine-targeted intervention reshapes the local hormonal growth network and further influences the host’s systemic immune landscape (Figure 3B).

### 4.3. Microbial-Derived Metabolites Influence the Efficacy of Immune Checkpoint Inhibitors via Antagonism of Endogenous Receptors

In recent years, immune checkpoint inhibitors (ICIs) have significantly improved the clinical prognosis of various oncological patients, although their clinical response rates remain contingent upon the homeostasis of the gut microbiota. Large-scale real-world cohort analyses demonstrate that in patients with head and neck squamous cell carcinoma or non-small cell lung cancer receiving PD-1/PD-L1 inhibitors, the administration of broad-spectrum antibiotics disrupts the microbial architecture. Such perturbations, exemplified by the dysregulation of the Firmicutes to Bacteroidetes ratio, are closely associated with shortened overall survival and an increased risk of mortality [68,69]. Within this paradigm, the crosstalk between sex hormone signaling and the microbiota constitutes a pivotal regulatory axis for immunotherapeutic efficacy. Preclinical models of colorectal cancer indicate that antibiotic treatment results in accelerated tumor progression and immunosuppression in male mice, a phenomenon linked to systemic alterations in androgen levels and shifted infiltration patterns of Th17 and myeloid cells within colonic tissues following microbial depletion [33,70]. Recent mechanistic studies further elucidate the biochemical interactions between microbial metabolites and host hormone receptors within the TME. On one hand, endogenous androgen receptor (AR) signaling in T cells directly represses the transcriptional program of interferon-γ (IFN-γ), thereby constraining the anti-tumor T-cell responses elicited by anti-PD-1/PD-L1 therapies [13]. On the other hand, integrative multi-omic analyses suggest that secondary bile acids, generated through microbe-mediated structural modifications by specific gut taxa, can traverse the intestinal barrier into systemic circulation and competitively antagonize host AR signaling within the TME. This microbial metabolite-based endocrine blockade alleviates AR-mediated immunosuppression, subsequently enhancing the anti-tumor efficacy of ICIs [12]. These findings underscore that the gut microbiota, by simultaneously modulating the endocrine axis and immune responses through its metabolites, serves as a critical determinant of ICI clinical outcomes. To achieve a more focused understanding of PD-1/PD-L1 regulation, it is essential to consider the multifaceted impact of microbial metabolites on checkpoint stability and expression. Recent evidence highlights that SCFAs, such as butyrate and propionate, can remodel the TME by regulating the ubiquitin-proteasome system (UPS) and specific E3 ligases, which are critical for the post-translational modification and degradation of immune checkpoint proteins [71]. Furthermore, these metabolites modulate the cytokine milieu (e.g., IFN-γ and IL-12), subsequently influencing the JAK/STAT signaling pathway to suppress PD-L1 expression on tumor-associated macrophages and cancer cells [72]. At the epigenetic level, SCFAs like pentanoate and butyrate function as potent class I HDAC inhibitors. By coordinating with the mTOR metabolic sensor, they significantly enhance the production of effector cytokines (IFN-γ and TNF-α) in cytotoxic T lymphocytes, thereby reversing immune evasion [73]. Integrating these microbial-molecular signatures through AI-driven analytics offers a promising avenue for advancing precision immunotherapy and optimizing individualized treatment strategies [71]. Taken together, these bidirectional interactions characterize the core molecular network of drug resistance evolution under the microecology–endocrine–immune tripartite axis, driven by three synergistic pathways: (i) enzymatic reprogramming of systemic drug exposure and inactivation via microbial metabolic networks (e.g., GUS and reductive pathways for Enzalutamide); (ii) treatment pressure-induced ecological succession that facilitates bypass hormone synthesis (e.g., 11-oxyandrogens); and (iii) microbial metabolite-mediated fine-tuning of immune checkpoint signaling and alternative purine signaling (e.g., the Inosine-A2AR axis). Collectively, these mechanisms provide a comprehensive molecular roadmap for deciphering therapeutic failure in hormone-dependent malignancies (Figure 3C).

Building upon the aforementioned biochemical interactions and microbial succession processes, pharmacomicrobiomics offers a novel perspective for deciphering the resistance mechanisms inherent in hormone-dependent malignancies. The gut microbiota has evolved in scientific understanding from a mere metabolic carrier to a critical determinant influencing the clinical responsiveness of anti-tumor pharmacological agents. Through mechanisms such as microbe-mediated structural modifications, the microbiota actively modulates systemic drug exposure, the onset of adverse toxicities, and the activation of anti-tumor immune responses. To provide a more intuitive representation of the clinical observations and mechanistic evidence in this field, we have systematically organized and synthesized the bidirectional regulatory networks between mainstream endocrine and immunotherapeutic agents and the gut microbiota (Table 3).

## 5. Clinical Strategies for Targeting the Gut Microbiota in Hormone-Dependent Malignancies

### 5.1. Precision Nutritional Interventions Targeting Microbial Fermentation to Regulate Systemic Metabolism

The ultimate objective of mechanistic research is to facilitate tangible clinical benefits. As the interactive network of the microbiota-endocrine-immune axis becomes increasingly defined, the focus in this field is shifting from the observation of pathological phenomena toward targeted, ecology-based interventions. To address the complex mechanisms of resistance evolution in hormone-dependent tumors, researchers are dedicated to constructing multimodal clinical intervention systems that encompass dietary nutrition, natural products, exercise prescriptions, and live biotherapeutics. These efforts aim to modulate the host anti-tumor microenvironment from a metagenomic dimension. Within this intervention framework, diet emerges as a first-line translational strategy, representing the most direct and highly adherent environmental factor for modulating the gut microbiota. In hormone receptor-positive malignancies, the coupling of specific phytonutrient intake with microbial metabolism demonstrates significant potential for intervening in disease progression.

The controversy surrounding soy isoflavones and the subsequent elucidation of their biochemical mechanisms provide a quintessential case study. Traditional perspectives have questioned the potential pro-proliferative risks of these compounds in breast cancer patients. However, microbial metabolic kinetic studies reveal that the ultimate biological effects of soy isoflavones are highly contingent upon the capacity of specific intestinal taxa, such as the genus *Slackia*, to convert them into equol through microbe-mediated structural modifications [74,75]. In subpopulations possessing this metabolic potential, equol exhibits the characteristics of a selective estrogen receptor modulator (SERM), enabling it to competitively antagonize the oncogenic signaling of endogenous estrogens. Conversely, patients lacking these specific microbial cohorts do not derive comparable biochemical benefits [75]. This nutritional intervention model, based on microbial metabolic phenotyping, provides an evidence-based foundation for the formulation of precision clinical nutritional prescriptions.

Compared to isolated components, whole-food dietary interventions yield broader systemic metabolic regulatory effects. Preclinical intervention models confirm that the regular consumption of pinto beans, which are rich in specific dietary fibers and antioxidants, can ameliorate gut dysbiosis driven by high-fat diets [76]. Intervention with pinto beans not only partially restores the ecological niche of SCFA-producing bacteria but also effectively reduces systemic levels of pro-inflammatory cytokines. Beyond mitigating this metabolic-inflammatory feedback loop, the microbiota-diet interaction further modulates the systemic bioavailability of sex hormones. Specifically, fiber-induced SCFAs can enhance the hepatic synthesis of sex hormone-binding globulin (SHBG), which serves as a critical buffer to sequester free estrogens and androgens, thereby neutralizing the hormonal drivers that promote abnormal endometrial and mammary proliferation [53,76]. While dietary-mediated SHBG modulation offers a promising non-pharmacological strategy, it should be noted that the clinical evidence regarding specific dietary patterns remains largely based on observational studies. Further verification through rigorously designed, large-sample randomized controlled trials (RCTs) is needed to define the optimal dietary implementation for specific cancer types [77].

### 5.2. Traditional Chinese Medicine Formulae Regulate the Microbiota via Multitargeted Network Mechanisms

Traditional Chinese Medicine (TCM) encompasses a diverse array of natural bioactive constituents. Within the analytical framework of modern systems biology, specifically the microbiota-metabolism-enzyme axis, natural products exemplified by TCM formulae have been validated as potent microbial modulators, offering multi-targeted strategies for intervening in the tumor microenvironment.

In the adjuvant treatment of breast and colorectal cancers, the classical TCM preparation Xihuang Pill demonstrates non-cytotoxic systemic regulatory effects. Integrated immunometabolic and omics analyses reveal that Xihuang Pill modulates the intestinal microbial metabolic network. One key mechanism involves mitigating the enrichment of opportunistic pathogens and intervening in steroid hormone biosynthetic pathways, thereby restoring the Treg/Th17 cell balance in both peripheral blood and the tumor-infiltrating microenvironment [78,79,80]. This microbiota-mediated systemic immunomodulation establishes an immunological foundation for inhibiting tumor invasion. Furthermore, in the investigation of metabolic complications and inflammatory microenvironments, natural formulations such as Ziwan granules have demonstrated the capacity to regulate lipid metabolism and the host hormonal axis [81]. By influencing primary and secondary intestinal metabolites through microbe-mediated structural modifications, these formulations modulate host lipid transport and energy metabolism pathways. This intervention ameliorates the pro-oncogenic environment of hyperestrogenism derived from central obesity, providing a candidate strategy for the early intervention of obesity-related hormone-dependent malignancies, such as endometrial cancer [48,81].

### 5.3. Host Genetic Background Modulates the Regulatory Effects of Exercise Interventions on Endocrine and Microbial Systems

Physical exercise interventions are being increasingly integrated into oncological rehabilitative frameworks as systemic modulators of endocrine homeostasis. However, the modulation of the microbiota-endocrine axis by exercise exhibits pronounced inter-individual heterogeneity, with the host genetic background exerting a fundamental restrictive influence. A recent genome-wide association study (GWAS) involving 16,017 individuals demonstrated that the host FUT2 genotype shapes a distinct “intestinal molecular landscape” by modifying mucosal layer components and bile acid profiles, thereby genetically pre-defining the thresholds for microbial responses to lifestyle interventions [82].Within clinical cancer cohorts, investigations have identified the mediating role of the apolipoprotein E (APOE) genotype in the field of exercise microbiology [83]. Under standardized exercise protocols, prostate cancer patients harboring divergent APOE alleles manifest differential microbial and endocrine response patterns. Evidence suggests that the interplay between exercise and the APOE genotype is associated not only with shifts in the α-diversity of the gut microbiota but also with fluctuations in systemic testosterone levels and associated cognitive functional indices [83]. Furthermore, this genetic constraint potentially impacts the efficiency of exercise-induced remodeling of the tumor immune microenvironment, where specific genetic backgrounds may limit the biosynthesis of exercise-induced anti-inflammatory metabolites, such as butyrate and other short-chain fatty acids. Crucially, the clinical examination of these interventions reveals that physical activity remodels the microbial-bile acid axis to overcome adaptive resistance. Regular exercise promotes the enrichment of taxa capable of converting primary bile acids into secondary metabolites like UDCA, which competitively antagonize hormone receptors to re-sensitize tumor cells to endocrine therapies. This complex interaction, encompassing genetic background, exercise-induced metabolic remodeling, and microbial response, underscores the clinical value of personalized exercise prescriptions in circumventing the evolutionary bypasses of treatment resistance [82,83]. Nonetheless, the translation of exercise-induced microbial remodeling into precision oncology necessitates further longitudinal studies. In particular, the synergistic effects of prebiotics and probiotics as adjuvants to lifestyle interventions require more systematic examination to determine their exact clinical benefits in restoring endocrine homeostasis.

### 5.4. Live Biotherapeutics Facilitate Clinical Translation of Microbial Interventions via Active Colonization Pathways

Dietary, herbal, and exercise interventions essentially represent passive modulations of pre-existing microbial networks. To overcome the resistance barriers inherent in endocrine and immune-targeted therapies, clinical translation is advancing toward active colonization strategies based on Live Biotherapeutics (LBPs). Addressing microbial dysbiosis induced by chemotherapy or antibiotic exposure, fecal microbiota transplantation (FMT) has demonstrated significant promise in combined immunotherapy for metastatic malignancies, such as melanoma and colorectal cancer, by enhancing immune responses and circumventing ICI resistance [84].

Regarding tamoxifen-induced hepatotoxicity or resistance to targeted agents, next-generation precision microbial therapies are focused on developing obligate engineered probiotic consortia with specific metabolic deletions, such as inactivated GUS enzymes, or utilizing highly purified preparations of *Akkermansia muciniphila* for directed colonization [59,61]. These live biotherapeutic interventions, designed to disrupt the hormonal metabolic loop by modulating microbe-mediated structural modifications, signify that the comprehensive management of hormone-dependent malignancies is expanding into an active ecological dimension. To integrate these multidimensional explorations into an actionable clinical framework, we have systematically summarized the underlying mechanisms and clinical translational evidence for multimodal intervention strategies, encompassing precision nutrition, natural products, exercise prescriptions, and live biotherapeutics (Table 4).

## 6. Discussion

The progression of hormone-dependent malignancies is recognized as a systemic process involving macro-ecological dynamics and micro-molecular communication. The preliminary elucidation of the microbiota-endocrine-immune axis provides a novel systems biology perspective for overcoming resistance to targeted therapies. However, current research is at a critical juncture, transitioning from phenomenological observation toward mechanistic precision intervention. Effectively translating microbial science into clinical oncological strategies necessitates addressing multi-faceted scientific challenges at both the fundamental and translational levels.

Over the past decade, observational studies utilizing 16S rRNA amplicon sequencing have delineated extensive dysbiosis profiles for breast cancer [6,85,86], prostate cancer [7,9,11], and gynecological malignancies [4,87]. Nevertheless, this technological paradigm harbors inherent limitations, as correlations in taxonomic abundance do not equate to causality in metabolic function. The gut microbiota exhibits high functional redundancy. For instance, GUSs, which dictate estrogen reabsorption, are widely distributed across phylogenetically unrelated bacterial phyla [88]. Conversely, steroid cleavage pathways driving castration resistance may be sequestered within ultra-low-abundance strains, such as *Clostridium scindens* [29]. Consequently, future research must shift from taxonomy toward functional metabolism and spatial dynamics. This transition requires integrating deep metagenomic assembly with AI-based structural biology predictions, such as modeling the 3D structures and substrate-binding sites of uncharacterized microbial enzymes [88], to precisely identify strain-level specialized enzymatic functional clusters. Furthermore, genetic causal inference methods, such as Mendelian Randomization (MR), must be introduced to exclude confounding factors and establish causality within the host genetic context [89,90,91]. The observational associations described above, while informative, do not establish whether microbiota alterations are a cause or consequence of cancer progression and treatment resistance. Direct causal evidence derives from three complementary approaches. First, germ-free and gnotobiotic mouse models provide functional validation. Colonization of germ-free mice with gut microbiota from anti-PD-1 responder versus non-responder melanoma patients reproducibly transfers the responsiveness phenotype, with responder-microbiota mice exhibiting a pro-inflammatory myeloid signature that correlates with improved ICI efficacy [92]. Similarly, *Clostridium butyricum* enhances anti-PD-1 efficacy in orthotopic colorectal cancer models, with its tumor-suppressive effect verified in AOM/DSS-induced CRC and germ-free mice [93]. Second, randomized clinical trials have directly tested whether microbiome modulation alters clinical outcomes. The phase 2 TACITO trial—the first randomized, double-blind, placebo-controlled study evaluating FMT from complete ICI responders—demonstrated that FMT significantly improved progression-free survival in advanced metastatic renal cell carcinoma patients receiving pembrolizumab and axitinib [94]. The phase 2 FMT-LUMINate trial further showed that healthy donor FMT combined with anti-PD-1 achieved an objective response rate of 80% in non-small cell lung cancer and 75% in melanoma, with metagenomic sequencing confirming that loss of deleterious baseline bacterial species was required for therapeutic benefit [95]. Third, MR analyses leverage genetic variants as instrumental variables to overcome confounding and reverse causality. A two-sample MR study found that phylum and class Actinobacteria mediate 29% and 21%, respectively, of the BMI–colorectal cancer risk relationship, collectively accounting for 50% of the obesity-driven CRC risk [96]. Taken together, this multi-level causal evidence—from gnotobiotic models, randomized FMT trials, and genetic inference—collectively establishes that the gut microbiota can actively influence cancer progression and therapeutic response, rather than merely correlating with these processes.

Beyond the microbiota-endocrine-immune axis, non-hormonal secondary metabolites, including lactate [97], asparagine [98], and gut-derived TMAO [66], serve as critical messengers remodeling the tumor metabolic and immune microenvironment. These must be integrated into multi-omic predictive models, such as the iCEMIGE model [99], to construct systemic molecular interaction topologies. While this review primarily deciphers the microbiota-endocrine-immune axis, hormonal signaling represents only one dimension of the multifaceted inter-organ communication driving oncogenesis. It is essential to integrate non-hormonal pathways, particularly programmed cell death and epigenetic reprogramming, into the broader framework of tumor fate determination. Emerging evidence indicates that microbial metabolites can directly modulate cell death resistance; for instance, the tryptophan metabolite IDA suppresses ferroptosis in colorectal cancer cells via the AHR-ALDH1A3 axis [100]. Furthermore, considering that cuproptosis is a recently identified copper-dependent death mechanism linked to the mitochondrial TCA cycle [101], future research should investigate whether microbial-driven remodeling of host energy metabolism constitutes a novel bypass to trigger this unique death mode.

A critical barrier to the clinical translation of fundamental microbial research lies in the limitations of current animal models, such as germ-free mice and FMT models, which fail to fully simulate the complexity of the human exposome and cross-species disparities in core bile acids. Patients are persistently exposed to multidimensional environmental factors. For example, lipid metabolism disorders induced by host obesity not only alter the basal intestinal ecology but also drive pathological elevations in systemic estrogen exposure, creating a vicious cycle between metabolism and endocrinology [80,102]. In real-world settings, the occult intake of artificial sweeteners [103], circadian rhythm disruption [104], and regional variations in dietary fiber intake [105] significantly perturb microbial homeostasis. Furthermore, the systemic nature of cross-organ microbial axes increases the complexity of intervention. Dysbiosis is associated not only with systemic endocrine-metabolic abnormalities [106] but also with distal biochemical communication, where microbial metabolites traverse mucosal barriers to interact with the thyroid [79], reproductive tract, and endometrial microbiota [55,107,108,109]. Recent multicenter clinical evidence confirms that patients with hormone-dependent tumors exhibit highly consistent coordinated dysbiosis across anatomical sites [15,47,51]. Therefore, future longitudinal cohort designs must incorporate long-term dietary patterns [110] and the interaction between host genetic backgrounds, such as APOE, and exercise interventions [83] into multivariable-adjusted models to restore the true operational logic of the microbiota within the complex organism.

At the clinical intervention level, the complexity of pharmacomicrobiomics presents severe challenges to translation. One aspect is the context-dependency of live probiotic microbes. For instance, *Akkermansia muciniphila* has been identified as a beneficial taxon enhancing the efficacy of ICIs in various studies. However, in the specific inflammatory context of colorectal cancer, it may transition into an opportunistic pathogen by excessively degrading intestinal mucins, thereby promoting tumor evolution [111]. Conversely, indigenous host microbiota exhibits significant colonization resistance. Following exposure to broad-spectrum antibiotics [69,81,112] or sustained selective pressure from endocrine drugs [113,114], the microbial architecture in cancer patients often remains in a state of structural collapse. This hinders the long-term engraftment and stable efficacy of individual probiotic supplements or FMT [84]. Moreover, the bidirectional metabolic interaction between endocrine agents and the microbiota can lead to the substantial depletion of essential protective metabolites, such as HDCA, triggering severe gastrointestinal and hepatic toxicities [61] and unpredictable therapeutic fluctuations [115,116]. To achieve precision intervention of the host-microbe holobiont, future clinical translation must rely on rigorously designed, large-scale prospective randomized controlled trials, such as the TOLERANT [117], PROBES [118], and G-DEFINER [119] cohorts. Next-generation microbial intervention strategies are expected to evolve from non-specific live biotherapeutics toward precision molecular interventions. These include the development of postbiotic molecular replacement therapies. To achieve precision molecular intervention, future research must prioritize the role of microbiota-derived extracellular vesicles (EVs) as key interkingdom communicators. These vesicles act as long-range shuttles that transport effector molecules to distal organs, modulating host signaling and immune homeostasis with the potency of postbiotics [120,121].

## 7. Conclusions and Future Directions

Hormone-dependent malignancies are increasingly recognized as products of disrupted biochemical communication between the host and the symbiotic microbiota. The gut microbiota, acting as an extrinsic endocrine hub and metabolic reactor, modulates steroid hormone turnover and immune microenvironment remodeling through specialized enzymatic networks and post-translational modification pathways (e.g., UPS-mediated degradation). Given the inherent complexities of the human exposome and microbial colonization resistance, the evolution of therapeutic strategies involves transitioning from isolated host pathways toward the systemic stabilization of the host-microbe holobiont.

Future investigations are expected to pivot from descriptive taxonomic correlations to causal mechanistic dissection. Establishing functional causality through gnotobiotic validation and Mendelian Randomization represents a strategic approach to neutralizing environmental confounders. This framework provides robust evidence for clinical decision-making. Furthermore, high-resolution metagenomic assembly and structure-based enzymatic modeling provide a foundation for resolving strain-level metabolic flux. Translational efforts are evolving from empirical microbial modulation toward precision molecular interventions targeting the microbiota-liver-tumor axis. The development of small-molecule inhibitors for specific microbial enzymes, postbiotic replacement therapies, and extracellular vesicle-based delivery systems defines a frontier in next-generation oncology. By breaking the biochemical codes governing microbiota-endocrine communication, these approaches are poised to circumvent existing resistance barriers. Ultimately, this systemic strategy will optimize the comprehensive clinical management of hormone-dependent malignancies.

## Figures and Tables

**Table 1:** Gut microbiota-associated sex hormone metabolic networks and core enzymatic basis.

Functional Compartment	Substrate	Core Product	Key Enzyme(s)	Representative Bacteria	Biological Significance & Clinical Phenotypes	References
Estrobolome	Conjugated estrogens (E1-3-G, E2-17-G)	Free estrogens (E1, E2)	GUS isoforms (with Loop 1/FMN motifs)	Various prevalent gut commensals	Deconjugation & Reabsorption: Determines the set point of EHC efficiency and systemic active estrogen exposure.	[3,16,17,27,28]
Androbolome	Glucocorticoids (Cortisol, Prednisolone)	11-oxyandrogens (e.g., 1,4-androstadiene-3,11,17-trione)	Steroid-17,20-desmolase (DesAB)	*Clostridium scindens* (e.g., ATCC 35704)	Cross-Class Synthesis: Bypasses host endocrine axis to provide alternative androgenic stimulation; contributes to CRPC evolution.	[23,24,29]
Androbolome	Testosterone & circulating precursors	Dihydrotestosterone (DHT) & degraded metabolites	5α-reductase, Steroid dehydrogenases	*O. splanchnicus*, *P. nitroreducens*, *Thauera* sp.	Bidirectional Regulation & Degradation: Alters DHT/T ratio in females; or executes catabolic degradation/EHC interruption to deplete the androgen pool.	[18,30,31]
Progesta/Corticobolome	Glucocorticoids (Corticosterone, Cortisol)	Reduced metabolites (e.g., 5α-DHF)	Oxidoreductases, OsrABC complex	*C. scindens*, *C. steroidoreducens* and other gut commensals	Stress Network Crosstalk: Remodels host HPA axis outputs under acute immune stress, regulating systemic immune and stress homeostasis.	[20,21,22,24]
Progesta/Corticobolome	Glucocorticoids/Progestins	Progestins/Epipregnanolone (inactivated)	21-dehydroxylase, apmAB reductase	*Clostridium innocuum*, *G. pamelaea*, *E. lenta* and others	Bypass Transformation & Inactivation: Mediates cross-class conversion into progestins; or directly inactivates progestins into low-activity neurosteroids.	[25,26]
Redox Networks	Primary bile acids & steroid intermediates	Secondary bile acids (e.g., DCA) & reduced backbones	7α-HSDH, 3β/5β-reductase	*C. innocuum*, *H. hathewayi*	Basal Detoxification Hub: Links host lipid metabolism with local inflammation, maintaining systemic endocrine defense.	[19,32]

**Table 2:** Summary of Core Microbiota-Endocrine-Immune Mechanisms Across Different Cancer Types.

Cancer Type	Core Mechanism	Key Molecules/Biological Events	Clinical & Translational Significance	References
Breast Cancer	Estrobolome activation & immune remodeling	GUS enzymes reactivate circulating estrogens, driving ERα proliferation; high estrogen induces CD8+ T cell exhaustion and Tregs recruitment; *Veillonella* mediates aromatase inhibitor resistance. Validated by germ-free (GF) models.	Predicts long-term efficacy of endocrine drugs; Linkage between postmenopausal estrobolome shifts and malignancy; offers novel pathways to intervene in “cold tumor” microenvironments.	[1,3,27,28,39,40,49,50]
Prostate Cancer	Androgen bypass synthesis; Inter-kingdom crosstalk (e.g., PCOS-like excess)	Specific taxa (e.g., *C. scindens*) bypass ADT via de novo 11-oxyandrogen synthesis; alternative androgens induce M2 macrophage polarization (CD206+, IL-10 secretion); bile acid-mediated AR antagonism; validated by FMT from CRPC patients into gnotobiotic models.	Uncovers gut-derived drivers of castration resistance (CRPC); provides predictive biomarkers for macrophage reprogramming and immune checkpoint blockade.	[13,14,23,29,42,43]
Endometrial Cancer	Metabolic-endocrine-epigenetic axis (Obesity context)	Obesity-induced SCFA depletion; altered histone acetylation; LPS-driven PI3K/AKT oncogenic pathways; high free-estrogen synergy.	Supports genetic causal inference (Mendelian Randomization) for SCFA-mediated protection; clarifies synergy between hyperestrogenism and multi-site co-dysbiosis.	[15,44,45,46,47,48,51,52,53,54]
Ovarian Cancer	Gut-vaginal axis & TME remodeling	Microbial-derived metabolites (e.g., LPS, SCFAs) regulate oncogenic pathways, interfering with epithelial–mesenchymal transition (EMT) and mediating immune escape of the epithelial barrier.	Provides integrated multi-omics biomarkers for early screening and microbiota intervention in ovarian cancer.	[55,56,57]
Systemic Tumors (e.g., CRC)	Sex hormone-mediated immune dimorphism	Estrogen enhances anti-tumor T cell responses and reduces gut inflammation. Secondary bile acids act as active inter-kingdom messengers (competitive AR antagonism), relieving androgen-mediated systemic immunosuppression.	Perfectly explains sex disparities in immunotherapy response; offers novel targets for overcoming immune resistance via metabolites.	[12,34,35]

**Table 3:** Bidirectional Regulatory Networks between Endocrine/Immune Targeted Drugs and Gut Microbiota.

Therapeutic Agent	Target Cancer Type	Key Microbial & Metabolic Events	Pharmacodynamic & Clinical Consequences	References
Tamoxifen	Breast Cancer	Microbial GUS mediates prodrug deconjugation; drug induces gut dysbiosis (e.g., *Blautia* enrichment).	Causes inter-individual variability in active metabolite exposure; HDCA depletion interrupts hepatic FXR signaling, inducing hepatotoxicity.	[2,3,60,61]
Aromatase Inhibitors (AIs/AET)	Breast Cancer	Prolonged drug pressure decreases α-diversity; specific taxa (e.g., *Veillonella*) are enriched in resistant patients.	Specific microbial reshaping strongly correlates with increased recurrence risk and shortened progression-free survival (PFS).	[1,62,63]
Enzalutamide/Abiraterone	Prostate Cancer	Microbial reductive inactivation (for Enzalutamide); enrichment of *Akkermansia muciniphila* and 3β-HSD-mediated transformation (for Abiraterone).	Decreases systemic AUC; accompanied by upregulated serum inosine, or provides alternative androgenic fuel to promote resistance.	[14,58,59]
Androgen Deprivation Therapy (ADT/ARSI)	Prostate Cancer	Microbial desmolases bypass synthesis of 11-oxyandrogens; TMAO activates the p38/HMOX1 signaling pathway.	Provides non-gonadal hormone fuel to tumor cells, driving the onset and progression of castration-resistant prostate cancer (CRPC).	[14,65,66,67]
Immune Checkpoint Inhibitors (Anti-PD-(L)1)	Pan-cancer/Colorectal Cancer	SCFA-mediated (Butyrate/Pentanoate) epigenetic and UPS regulation; microbial BAs competitively antagonize host AR signaling.	Modulates PD-L1 stability via UPS degradation; relieves AR-mediated immunosuppression, enhancing anti-tumor T cell responses.	[12,13,71,72,73]

**Table 4:** Multi-modal Microbiome Intervention Networks and Translational Evidence.

Intervention Dimension	Specific Strategy/Agent	Core Microbiota Mechanisms Supported by Evidence	Clinical Outcomes & Disease Targets	References
Precision Nutrition	Soy Isoflavones	Relies on specific taxa (e.g., *Slackia* sp.) to ferment precursors into active equol.	Equol acts as a SERM, competitively blocking potent estrogen oncogenic signals (in subsets with metabolic capacity).	[74,75]
Precision Nutrition	Pinto Beans	Specifically restores the niche of SCFA-producing bacteria, reducing systemic pro-inflammatory cytokines.	Blocks the metabolic-inflammatory loop, attenuating proliferative signals in endometrial and breast epithelia.	[53,76]
TCM & Natural Products	Xihuang Pill	Reconstructs microbiota metabolic networks and intervenes in steroid synthesis pathways.	Reverses pathobiont enrichment, restores Treg/Th17 homeostasis in blood and TME, and inhibits tumor invasion.	[78,79,80]
TCM & Natural Products	Aster tataricus extract	Intervenes in primary/secondary metabolite pools, regulating host lipid transport and energy metabolism.	Weakens the high-estrogen oncogenic environment derived from central obesity (e.g., in endometrial cancer).	[48,81]
Exercise & Host Genetics	Genotype-targeted Exercise Prescription (FUT2, APOE)	Host genetics shape the intestinal molecular landscape (e.g., via mucins and bile acids) to pre-define microbial response thresholds; exercise-induced trajectories are constrained by specific alleles.	Modulates α-diversity and upregulates SHBG to reduce systemic testosterone and associated cognitive indices (e.g., in prostate cancer patients).	[82,83]
Live Biotherapeutics	Fecal Microbiota Transplantation (FMT)	Comprehensively reconstructs collapsed niches due to chemo/antibiotics, restoring the metabolic-immune axis.	Reverses immunosuppression, overcoming immune checkpoint blockade (ICB) resistance in metastatic cancers (e.g., CRC).	[84]
Live Biotherapeutics	Engineered/Purified Bacteria	Target-specific blockage or supplementation of metabolic pathways (e.g., GUS-KO strains, purified *A. muciniphila*).	Eliminates tamoxifen-induced hepatotoxicity; reshapes the substrate pool to overcome endocrine therapy resistance.	[59,61]

## Data Availability

No new data were created or analyzed in this study.
